# Technical specifications for ethics review of human stem cell research

**DOI:** 10.1111/cpr.13556

**Published:** 2023-10-12

**Authors:** Yaojin Peng, Aijin Ma, Zhenyu Xiao, Jie Hao, Ruohan Feng, Changlin Wang, Baoyang Hu, Peijun Zhai, Ka Li, Lei Wang, Jiani Cao, Peng Xiang, Yu Zhang, Jun Wei, Xiaolong Wang, Boqiang Fu, Qubo Chen, Qiang Wei, Hengjun Gao, Jianwei Lv, Xing Liu, Yonghui Ma, Tao Cheng, Xiaoru Huang, Yeyang Su, Haihong Zhang, Qi Zhou, Tongbiao Zhao

**Affiliations:** ^1^ State Key Laboratory of Stem Cell and Reproductive Biology, Institute of Zoology, Chinese Academy of Sciences Beijing China; ^2^ Beijing Institute for Stem Cell and Regenerative Medicine Beijing China; ^3^ University of Chinese Academy of Sciences Beijing China; ^4^ Institute for Stem Cell and Regeneration, Chinese Academy of Sciences Beijing China; ^5^ Chinese Society for Stem Cell Research Shanghai China; ^6^ Beijing Technology and Business University Beijing China; ^7^ School of Life Science, Beijing Institute of Technology Beijing China; ^8^ National Stem Cell Resource Bank, Institute of Zoology, Chinese Academy of Sciences Beijing China; ^9^ China Stem Cell and Regeneration Collaborative Innovation Platform Beijing China; ^10^ China National Institute of Standardization Beijing China; ^11^ China National Accreditation Service for Conformity Assessment Beijing China; ^12^ Institute of Clinical Science, Zhongshan Hospital Fudan University Shanghai China; ^13^ Center for Stem Cell Biology and Tissue Engineering, Key Laboratory for Stem Cells and Tissue Engineering, Ministry of Education Sun Yat‐Sen University Guangzhou China; ^14^ Zephyrm Biotechnologies Co., Ltd Beijing China; ^15^ China National Research Institute of Food and Fermentation Industries Beijing China; ^16^ National Institute of Metrology Beijing China; ^17^ State Key Laboratory of Dampness Syndrome of Chinese Medicine The Second Affiliated Hospital of Guangzhou University of Chinese Medicine Guangzhou China; ^18^ National Pathogen Resource Center, Chinese Center for Disease Control and Prevention Beijing China; ^19^ Tongji Hospital, School of Medicine Tongji University Shanghai China; ^20^ Institute of Digestive Disease, School of Medicine Tongji University Shanghai China; ^21^ Xiamen University Xiamen China; ^22^ Xiangya Hospital Central South University Changsha China; ^23^ State Key Laboratory of Experimental Hematology and National Clinical Research Center for Blood Diseases, Institute of Hematology and Blood Diseases Hospital, Chinese Academy of Medical Sciences & Peking Union Medical College Tianjin China; ^24^ Institute of Basic Medicine and Cancer, Chinese Academy of Sciences Hangzhou China; ^25^ Peking University Health Science Center Beijing China

## Abstract

The rapid advancement of human stem cell research and its expansion into emerging areas has resulted in an escalation of ethical challenges associated with these studies. As a result, there has been a corresponding increase in both the volume and complexity of institutional ethics reviews, coupled with higher expectations for the quality of the review process. In response to these challenges, this standard provides a comprehensive outline of the fundamental principles, content, types, and procedures of ethics review, specifically focusing on non‐clinical human stem cell research. Its purpose is to provide clear operational and procedural guidelines, as well as recommendations, for the ethics review of such studies. The document was originally published by the Chinese Society for Cell Biology on August 30, 2022. It is our hope that the publication of these guidelines will facilitate the integration of ethical considerations and evaluations in a structured manner throughout the entire process of stem cell research, ultimately fostering a healthy and orderly development of the field.

## 1 SCOPE

This document outlines the ethical principles, review content, review types, and review procedures for human stem cell research. It is applicable to the ethics review of non‐clinical research on human stem cells.

## 2 NORMATIVE REFERENCES

This document does not contain normative references.

## 3 TERMS AND DEFINITIONS

The following terms and definitions are applicable to this document.

### 3.1 Human biological material

Fresh or preserved biological samples obtained from the human body in a lawful and reasonable manner.

*Note*: Examples include human germ cell precursor cells, germ cells, fertilized eggs, embryos, voluntarily aborted fetal tissues, as well as somatic peripheral blood, bone marrow, fat, skin, umbilical cord, umbilical cord blood, and so on.

### 3.2 Human biological data

Biological feature information related to human biological materials themselves, as well as information generated using human biological materials.

*Note*: This includes details such as the type, quantity, storage location, and classification information of samples in the database, as well as sequencing data of genome, transcriptome, and proteome, and so on.

### 3.3 Human non‐human animal chimera

An organism created by introducing human cells into non‐human animal embryos or bodies, resulting in the integration of human cells into another species and their coexistence during a specific stage.

### 3.4 Study protocol

Document that describes the purpose, design, methodology, statistical considerations, and implementation of human stem cell research.

*Note*: The protocol typically includes the background and theoretical foundation of the study. It comprises both the original protocol and revised versions.

### 3.5 Informed consent

A process of fully informing the potential human biological material donors about various aspects that may influence their decision to donate. Following this, the donors voluntarily confirm their consent to donate their biological material for research purposes.

### 3.6 Non‐compliance/violation

Any deviations from the approved study protocol by the ethics review committee that have not been pre‐approved or are non‐compliant/contradictory to the relevant regulations and requirements of the ethics review committee.

### 3.7 Independent consultant

Experts in relevant ethics, law, specific scientific domains, or methodologies who are hired or appointed by the ethics review committee, as well as representatives of the community, patients, or specific interest groups, provide specialized opinions and suggestions to the ethics review committee within the authorized scope.

## 4 PRINCIPLES OF REVIEW

### 4.1 Beneficence

Consideration shall be given to the research project's potential benefits to science, society, and patients. It shall promote scientific progress in the field of stem cells and related areas, protect the rights and interests of individuals' life and health, and contribute to the welfare of human being.

### 4.2 Risk control

Consideration shall be given to whether the research project minimizes harm or threats to human biological material donors, related groups, laboratory animals, and surrounding ecological environments, and so on. The principles of laboratory animal welfare, including reduction, replacement, and refinement, shall be followed.

### 4.3 Respect for autonomy

Consideration shall be given to whether the study project fully respects and safeguards the autonomy of human biological material donors, including their right to make autonomous decisions. This includes respecting their right to make autonomous decisions regarding donation, participation in the research, specific types and content of participation, stages of involvement, extent of participation, and the right to withdraw from the study.

### 4.4 Adequacy and necessity

Consideration shall be given to whether the research project has sufficient scientific rationale and justification for its implementation. Stem cell‐related research projects involving human germ cells, fertilized eggs, embryos, aborted fetal tissues, and so on, shall assess the necessity of the experimental protocol and consider other feasible alternative approaches.

### 4.5 Fairness

Thorough consideration shall be given to the potential impacts of the research project on various groups in terms of public health, medical resources, and social benefits. The development and application of technologies and products shall benefit a broader range of groups. The fair and reasonable distribution of risks and benefits resulting from the research should be considered. The research and its outcomes should not stigmatize, discriminate against, or unfairly treat specific groups.

### 4.6 Scientific integrity

The research shall adhere to scientific principles; maintain independence, fairness, and transparency. The research shall comply with confidentiality requirements and remain free from personal interests or external factors, such as administrative interference.

### 4.7 Legality and compliance

Consideration shall be given to whether the research project complies with applicable laws, regulations, and other regulatory documents in the respective country. The project's objectives, content, source of human biological materials, facility environment, personnel qualifications, operational procedures, and publication of research results shall be reviewed to ensure compliance and prevent any instances of illegal or non‐compliant practices.

## 5 REVIEW CONTENT

### 5.1 General requirements

5.1.1 If newly collected human biological material is used for stem cell research, it shall be collected by institutions with the necessary legal qualifications and credentials. When stem cells are derived from human germ cells, fertilized eggs, embryonic tissues, or aborted fetal tissues, careful consideration shall be given to the psychological and physiological impacts on donors. Measures shall be taken to prevent the risks associated with the commercialization of human germ cells, fertilized eggs, embryonic tissues, and aborted fetal tissues. The source of stem cells should be traceable and verifiable through relevant material transfer agreements, data that confirms the identity of cell lines, informed consent forms, and other relevant documents.
5.1.2 If human biological material used in stem cell research is obtained from biobanks or commercial suppliers, it is necessary to confirm the legal qualifications and credentials of the biobanks or suppliers. The source of human tissues or cells provided by them, including the process of collecting and acquiring biological material from biobanks or suppliers, shall comply with ethical and regulatory requirements. It is important to ensure that the stem cells provided by biobanks or suppliers have obtained informed consent and ethics review approval, and that the proposed research aligns with the scope of the original donor's informed consent authorization. Additionally, a compliant human biological material transfer agreement should be in place.
5.1.3 If stem cells obtained from human germ cells, zygotes, embryos, aborted fetal tissues, or other sources are intended for research purposes, a comprehensive evaluation of the scientific objectives and study protocol shall be conducted. This evaluation is crucial to ensure the scientific rigour of the research and requires strong and valid scientific justifications. The assessment should also consider technical factors such as the level of technological advancement, operational complexity, and controllability, as well as ethical risks such as the likelihood and severity of ethical concerns and the availability of contingency plans to address these risks.
5.1.4 Given the involvement of human biological materials and data in stem cell research, it is essential to ensure that the storage, use, processing, transmission, sharing, and publication of human biological materials and related data, including stem cells, are carried out in an anonymous or de‐identified manner. A robust donor privacy protection system shall be established, and multiple layers of security measures should be implemented to safeguard the data, including the implementation of access permissions.

### 5.2 Review methods

5.2.1 Ethics review methods pertaining to stem cell research typically include expedited review and full board review. In special circumstances, it is advisable to conduct a peer review initially to thoroughly evaluate the scientific validity, rationale, and research value before proceeding with the ethics review.
5.2.2 The following research contents should undergo full board review:
  - The use of human germ cells, fertilized eggs, or embryos for in vitro research;
  - The utilization of human germ cells, fertilized eggs, or embryos to obtain stem cells or establish stem cell lines;
  - The in vitro culture of human embryos for research until the onset of primitive streak or up to 14 days after fertilization, whichever occurs first;
  - Research related to the in vitro culture of chimeric embryos formed by introducing human cells into non‐human embryos;
  - Research involving the introduction of human cells into non‐human embryos or foetuses and their subsequent gestation in a non‐human uterus;
  - Research involving the introduction of human stem cells into the nervous system or reproductive system of postnatal animal hosts;
  - Research related to the construction of cerebral organoids based on human stem cells;
  - Research related to the construction of non‐integrated or integrated embryo models based on human stem cells;
  - Other stem cell research projects that require full board review as suggested by the members of the ethics review committee.
5.2.3 For research projects that do not fall under the categories listed in 5.2.2 and involve minor modifications to approved study protocols without affecting the risk–benefit ratio of the research, expedited review can be conducted. However, expedited review shall be converted to full board review in the following situations: if the review results in a negative opinion, if there is disagreement among two committee members, or if a committee member proposes the need for full board review.
5.2.4 If the following research contents are identified during the review process, they shall not be approved through ethics review:
  - Human reproductive cloning;
  - The use of gametes differentiated from human stem cells for reproduction;
  - The use of embryo models constructed based on human stem cells for reproduction;
  - The use of genetically modified human embryos or gametes for reproduction;
  - Research involving the introduction of non‐human primate pluripotent stem cells or multipotent stem cells into human embryos;
  - Breeding human‐animal chimeras where there may be human germ cells;
  - Transferring human‐animal chimeric embryos into the uterus of a human or ape;
  - Other research that seriously violates social ethics, harms laboratory animal welfare, or undermines the welfare of human well‐being.

## 6 TYPES OF REVIEW

### 6.1 Initial review

6.1.1 Review of study protocol
  6.1.1.1 The key points to consider in the review of the study protocol shall include
    - The research holds scientific and societal significance.
    - The research objectives are clearly defined, well‐grounded, and scientifically valid.
    - The sample size is scientifically justified.
    - The evaluation criteria for safety and efficacy are reasonable.
    - The risk–benefit ratio of the research is reasonable.
    - Informed consent is reasonable.
    - Appropriate risk control and emergency response plans are in place.
    - A data security and monitoring plan is in place.
    - The qualifications, experience, time commitment of the researchers, as well as the personnel and equipment requirements, align with the experimental needs.
    - The research shall adhere to legal requirements, regulations, and pertinent national guidelines.
    - The presence of conflicts of interest in the research is assessed.
    - The presence of socially sensitive ethical issues in the research is assessed.
    - The publication of research results and the suitability of the publication method and timing are assessed.
    - Other essential aspects necessitating review are considered.
  6.1.1.2 In addition to the aforementioned review points, the initial review should give special attention to the presence of research content that is not permissible for ethical approval. In the case of stem cell research involving the utilization of human gametes, fertilized eggs, embryos, voluntarily aborted fetal tissues, and other sources, more stringent criteria should be applied. These criteria include considerations such as sample size determination, qualifications and experience of researchers, and other relevant aspects. For instance, if there is any genetic modification of human gametes or embryos for in vitro research, it is essential to ensure that the sourcing of human biological materials is legal and compliant. The research proposal should specify the minimum number of embryos or gametes to be used, and the researchers should possess appropriate expertise and have completed necessary training in gene modification and characterization. Additionally, the research institution shall possess the necessary infrastructure to support such research.
  6.1.1.3 Research endeavours that involve the generation of human embryos for the purpose of obtaining stem cells or establishing stem cell lines should satisfy the following conditions: the proposed research cannot be accomplished using existing human embryonic stem cell lines, and it is not feasible to acquire stem cells for research from voluntarily donated embryos that remain from in vitro fertilization treatments for infertility. The minimum number of embryos necessary for the research should be clearly defined. Researchers should possess appropriate expertise and have completed necessary training in the extraction, characterization, and cultivation of human embryonic stem cells. Additionally, the research institution shall possess the required infrastructure to support such research.
6.1.2 Points for reviewing informed consent
  6.1.2.1 When conducting research projects that involve the recruitment of human biological material donors, it is essential to obtain the informed consent of the donors prior to utilizing their biological materials and data for research purposes. The information provided during the informed consent process shall be comprehensive, clear, and easily understandable, without any form of coercion, deception, or undue influence.
    - The sufficient information disclosed in the informed consent process shall encompass not only the storage duration and location of the human biological materials, storage and confidentiality measures, potential treatment and applications, and whether they will be used for commercial purposes, but also the potential research objectives of the materials. This includes elucidating their potential use in generating human embryonic stem cells, induced pluripotent stem cells, and the establishment of other immortalized cell lines, embryos, or gametes.
    - The language employed in the informed consent process shall be readily comprehensible to the general public and aligned with local cultural customs. This will facilitate the understanding of potential biological material donors (or their guardians or legal representatives) and empower them to make voluntary decisions regarding the donation of their materials and their involvement in the research. This entails providing information on the specific type, content, stage, and extent of participation.
    - Donors shall be informed about their right to withdraw their donation of human biological materials and data at any time, with relevant personnel available for communication. Additionally, information regarding the timing and basic procedures for sample destruction should be provided.
  6.1.2.2 The process of obtaining informed consent shall be conducted in a reasonable manner. The individuals responsible for carrying out the informed consent process should possess knowledge of stem cell‐related research and undergo appropriate training to ensure the standardized implementation of informed consent procedures. It is crucial to avoid any conflicts of interest between the individuals conducting informed consent and the donors, unless there are sufficient justifications. In cases where researchers are involved in the informed consent process, their roles and any potential conflicts of interest shall be disclosed. Furthermore, consultation services should be made available to potential donors upon their request prior to the collection of human biological materials and data. Sufficient time and opportunities should be provided to the donors by the individuals conducting informed consent to ask questions and engage in discussions regarding relevant information. The informed consent process should be continually improved, and the informed consent form should be revised to address the specific requirements of different stem cell research protocols.
  6.1.2.3 For research projects involving the use of human biological materials obtained from biobanks or commercialized suppliers, it shall be confirmed that the providers of these materials can provide documentation demonstrating compliance with Chinese laws, regulations, and ethical standards regarding the acquisition process. These documents may include ethics review approvals and signed informed consent forms. The informed consent form should explicitly allow for the use of donated materials for scientific research, and it shall be ensured that the intended research falls within the scope authorized by the original consent. In cases where the informed consent form includes clauses prohibiting the use of materials for the specific research project, it shall be confirmed that the intended research does not fall within that restricted scope. In situations where human biological materials have been stored but have not previously obtained informed consent, the ethics review shall assess whether researchers have the possibility of contacting the donors to obtain their informed consent specifically for the project.
  6.1.2.4 In the case of donors who lack civil capacity or have limited civil capacity, the informed consent process shall involve obtaining written consent from their guardian or legal representative. If obtaining written consent is not feasible, oral informed consent may be obtained in advance, but it is crucial to maintain procedural records and have third‐party witnesses present. When the guardian or legal representative provides consent on behalf of the donor, all relevant information shall be effectively communicated to the donor in a manner they can understand, and efforts should be made to have the donor sign the informed consent form.
6.1.3 Waiver of written informed consent
  A waiver of written informed consent refers to circumstances where the donor's signature on the informed consent form is not required under specific conditions. However, it is important to note that a waiver of written informed consent does not eliminate the need for an informed consent process. The donor shall still be provided with relevant information and give consent. The donor can provide consent through verbal or alternative means, but there should be documentation of the process and third‐party witnesses present. The ethics review committee is responsible for evaluating materials similar to informed consent forms, such as informational documents used to inform donors. The committee may also request researchers or individuals responsible for obtaining informed consent to provide written informational materials to the donors.
6.1.4 The ethics review committee has the authority to make decisions regarding the reviewed research activities, which may include approval, conditional approval with modifications, re‐review after modifications or disapproval.

### 6.2 Follow‐up review

#### 6.2.1 Annual/periodic follow‐up review

6.2.1.1 The frequency of annual/periodic follow‐up reviews should be determined by the ethics review committee during the initial review, taking into account the level of risk and duration of the research project. These reviews should be conducted at least once a year. Upon reviewing the research progress, the ethics review committee should reassess the risks and benefits of the study to determine if any changes to the frequency of follow‐up reviews are necessary.
6.2.1.2 The ethics review committee may remind researchers to submit applications for annual/periodic follow‐up reviews 1 month before the expiration date. The required documents for submission may include the application form for annual/periodic review, annual project reports (if applicable), and published articles (if applicable).
6.2.1.3 The review elements include the following:
  - The ethics review committee shall assess the ethical risks of the research based on the progress reports submitted by the researchers and review any changes that may impact the ethical risks of the study. If deemed necessary, the committee may provide suggestions for improvement.
  - If any changes to the research protocol occur during the study, the ethics review committee shall verify whether these changes have been submitted for review and obtained approval before implementation.
  - The ethics review committee shall verify if ethical incidents are promptly reported and appropriately handled, and closely monitor the reporting of unexpected ethical incidents. Based on the reported content, a re‐evaluation of the ethical risk–benefit ratio of the research should be conducted.
  - If any new information relevant to the research emerges that may affect the ethical risk–benefit ratio, the ethics review committee shall reassess the ethical risks based on the latest information.
6.2.1.4 After the review, the ethics review committee may make decisions such as approval, conditional approval with modifications, approval with modifications, termination, or suspension (with specified dates) of the research.
6.2.2 Review of amendments
  6.2.2.1 The ethics review committee shall review modifications or clarifications made to the approved research protocol, informed consent form, and other relevant documents and information related to the research. If significant modifications are made to the informed consent form concerning the donation of human biological materials and data, the donor's consent shall be obtained again. The revised protocol can be implemented only after receiving approval through the ethics review committee's review process.
  6.2.2.2 The required documents for submission include the application form for amendment ethics review and the corresponding supporting materials.
  6.2.2.3 The review elements include:
    - Reassessment of the ethical risks and benefits of the research.
    - Verifying if the amendment goes beyond the scope of consent initially provided in the informed consent form concerning the use of human biological materials. If there are modifications that exceed the initial consent, obtaining re‐consent is required.
  6.2.2.4 Following the review, the ethics review committee may decide on approval, approval with modifications, conditional approval for modifications, non‐approval, termination, or suspension (with specified dates) of the research.
6.2.3 Review of non‐compliance/protocol violation
  6.2.3.1 The ethics review committee shall review all instances of non‐compliance/protocol violation that arise during the implementation of approved research projects, including matters concerning the informed consent process.
  6.2.3.2 The necessary documents for submission comprise the application form for reviewing non‐compliance/protocol violation and relevant reports.
  6.2.3.3 The non‐compliance/protocol violation review includes the following considerations:
    - Assessing the ethical and social implications of the incident, considering its nature, scope, and severity. Analysing the consequences, including any undue negative effects on donors, related groups, and society, as well as potential violations of donors' right to informed consent.
    - Examining the impact of the non‐compliance/protocol violation incident on the scientific validity of the research, including its implications for data and result integrity and reliability.
  6.2.3.4 The opinions provided by the ethics review committee after the examination may include:
    - Allowing the research to continue;
    - Modifying the protocol and/or informed consent form;
    - Initiating an investigation into the non‐compliance/protocol violation incident;
    - Providing additional training for the researchers;
    - Assigning the researchers to work under the supervision of senior researchers;
    - Restricting the participation of researchers in the research;
    - Rejecting subsequent research applications from the involved researcher;
    - Obtaining informed consent again;
    - Temporarily suspending the approved research;
    - Terminating the approved research.
6.2.4 Suspension/early termination review
  6.2.4.1 The ethics review committee is responsible for reviewing applications from researchers to suspend or terminate research projects prior to their completion.
  6.2.4.2 The necessary documents for submission comprise the application form for reviewing the suspension/early termination and relevant reports.
  6.2.4.3 The review considerations include:
    - Assessing the reasons provided by the researchers for the suspension/early termination and evaluating the reasonableness of the procedures involved. Evaluating the subsequent arrangements for handling human biological materials and data after the study has been suspended/terminated.
    - Considering whether additional measures are necessary to safeguard the rights and interests of the donors, relevant groups, and society.
  6.2.4.4 After the review, the ethics review committee may provide the following opinions:
    - Agreement to the suspension/early termination.
    - Recommendations for implementing appropriate protective measures.
6.2.5 Study completion review
  6.2.5.1 It is the responsibility of the ethics review committee to review the research completion report.
  6.2.5.2 The necessary documents for submission comprise the application form for reviewing the study completion report and relevant reports.
  6.2.5.3 The review points include:
    - Assessing whether the actual ethical risks during the research exceeded the anticipated risks evaluated during the initial review, based on the information provided in the report.
    - For projects with risks that exceeded expectations, considering whether additional governance measures are required.
  6.2.5.4 After the review, the ethics review committee may provide the following opinions:
    - Agreement to the study completion.
    - Agreement to the study completion after implementing appropriate measures.

### 6.3 Re‐review

6.3.1 If the ethics review committee's opinion is “conditional approval with modifications” or “re‐review required after modifications,” the committee shall review the resubmitted protocol once the required modifications have been completed.
6.3.2 The necessary documents for submission consist of a description of the modifications and the revised materials.
6.3.3 The review considerations include the following:
  - The primary reviewer or assigned primary reviewer shall cross‐reference the resubmitted documents with the original review comments to ensure that the researchers have accurately understood and adequately addressed the ethics review committee's suggestions for modification. If necessary, further communication with the researchers shall be conducted by the ethics review committee.
  - For modifications where the ethics review opinion was “conditional approval with modifications,” the primary reviewer shall complete a re‐review worksheet. For modifications where the ethics review opinion was “re‐review required after modifications,” the primary reviewer shall complete a review form.
  - The ethics review committee shall carefully review any objections or clarifications provided by the researchers regarding the ethics review opinions and consider their reasonable perspectives. The ethics review opinions shall be grounded in recognized ethical principles, clearly state the rationale for the decision, and foster comprehensive communication and exchange of views with the researchers.

## 7 REVIEW PROCEDURE

### 7.1 Application and acceptance of ethics review

7.1.1 The ethics review committee should establish accessible channels, such as a dedicated website or relevant links, to facilitate researchers' understanding of the specific requirements for the ethics review application and the committee's workflow. This may include a checklist of submission documents, ethics review application form, research proposal outline, and informed consent form templates.
7.1.2 The secretary is responsible for verifying the completeness of the submitted documents based on the checklist.
7.1.3 The secretary should conduct a preliminary review of the research proposal and informed consent form to ensure their completeness before further processing.
7.1.4 The secretary is responsible for confirming the completeness of all submitted documents and then submitting them to the chairperson or vice‐chairperson to determine the appropriate review method.
7.1.5 Once the review acceptance decision is made, the secretary shall promptly notify the researchers about the acceptance of their ethics review application.
7.1.6 The secretary is responsible for maintaining a complete record of all written documents in the pending review file cabinet and submitting them to the ethics review committee for review at the designated time.

### 7.2 Full board review

7.2.1 Review preparation
  7.2.1.1 The ethics review committee is responsible for determining the minimum number of attendees required for a full board review, which should include at least two‐thirds of the total committee members. These attendees should consist of experts from various relevant fields such as life sciences, medicine, bioethics, or law. It is important to ensure gender diversity among the attendees. For the review of stem cell research, committee members shall possess the necessary expertise in the ethical evaluation of such research. The specific number of attendees will be determined based on the circumstances of each review, taking into consideration any potential conflicts of interest among the members. Members with conflicts of interest should voluntarily recuse themselves from the review process. Furthermore, the committee should ensure that the number of voting members meets the legal requirements. Independent consultants may be invited to attend the meeting and provide advisory opinions, although they will not participate in the voting process. The secretary of the committee should schedule the meeting time and location well in advance and promptly notify the committee members and independent consultants of the details.
  7.2.1.2 Each project should be assigned one or two primary reviewers who are selected based on their professional expertise, understanding of the ethical concerns involved, and familiarity with the social‐cultural context of the project. It is important to avoid assigning primary reviewers who have conflicts of interest with the project under review. The primary reviewers are responsible for conducting their review prior to the full board review process.
  7.2.1.3 The secretary of the ethics review committee is responsible for preparing the meeting agenda and distributing important documents to the committee members. These documents include the meeting notification, minutes from the previous meeting, meeting agenda, review forms, research proposal, informed consent form, and other relevant materials. To allow sufficient time for committee members to review the documents before the meeting, they shall be delivered to the attending committee members at least 3 days in advance. This ensures that committee members have ample time to familiarize themselves with the materials and come prepared for the meeting.
7.2.2 Review process
  7.2.2.1 Attendees are required to sign in upon arrival at the meeting.
  7.2.2.2 Typically, the chairperson presides over the meeting. However, if the chairperson is absent or has a conflict of interest with the review project, the vice‐chairperson or a designated member appointed by the chairperson assumes the role of the meeting chairperson.
  7.2.2.3 The chairperson announces the start of the meeting, reminds attendees of any conflicts of interest and recusal statements, and verifies that the attendance requirements and composition of attendees meet the legal standards.
  7.2.2.4 The meeting briefing includes the minutes from the previous meeting, a summary of the preliminary review results, and other relevant supporting materials.
  7.2.2.5 It is recommended for the principal investigator to present the research proposal and address any ethical issues raised in the proposal. They should be prepared to answer questions from the committee members. Alternatively, if feasible, the primary reviewer may present the proposal and related ethical issues initially, and the principal investigator can join in to answer additional questions from the committee members.
  7.2.2.6 The meeting chairperson asks the researchers, committee members with conflicts of interest with the project, and any other relevant individuals to leave the room during the discussion and voting.
  7.2.2.7 The primary reviewer presents their review opinions on the research proposal and informed consent form item by item.
  7.2.2.8 Committee members engage in comprehensive discussions on the proposal. The meeting chairperson summarizes the key points of the discussion and presents a motion for voting.
  7.2.2.9 The ethics review decision is made through voting at the full board review meeting. A decision is reached based on the majority opinion of more than half of the committee members present.
  7.2.2.10 The ethics review committee shall meet the following requirements when making the decision: ensuring the completion of application documents, having a sufficient number of attending committee members as required by law, following the review procedure, conducting a comprehensive review and extensive discussion of the review points, excluding applicants and committee members with conflicts of interest during the discussion and voting, and ensuring that absentee committee members are not represented by others during voting.
  7.2.2.11 Committee members complete the review opinion voting form, vote on the research proposal and informed consent, provide specific comments and suggestions in the appropriate fields, and sign their names and dates. The ethics review committee determines the frequency of follow‐up reviews based on the level of ethical risks involved in the project, which can be indicated through voting or reflected in the meeting minutes.
  7.2.2.12 The meeting chairperson announces the voting results.
  7.2.2.13 The secretary is responsible for taking meeting minutes, which should include the meeting time and location, attendees, meeting chairperson, meeting agenda, committee members' opinions and suggestions, voting results, and the name of the recorder.
  7.2.2.14 The record of each research proposal review generally includes the names of the researchers, opinions and suggestions from the primary reviewer, substantial discussions among the committee members, declarations and recusals of members with conflicts of interest, voting results, the number of committee members in favour, against, and abstaining, as well as specific modification suggestions, recommendations, and reasons for objections.
  7.2.2.15 After the meeting, the secretary summarizes the discussions and decisions in a concise and readable format, completes the meeting minutes within 1 week, and submits them to the chairperson or vice‐chairperson for review and approval.
  7.2.2.16 The secretary reports the meeting minutes in the next regular meeting.

### 7.3 Expedited review

7.3.1 For each project, the chairperson or vice‐chairperson is responsible for assigning one or two primary reviewers. In the case of follow‐up reviews and re‐reviews, it is preferable to have the original primary reviewer conduct the review.
7.3.2 The secretary collects the evaluation forms from the reviewers, compiles the review opinions, and prepares the necessary notification or approval letters.
7.3.3 When the primary review opinion is “approval”, the chairperson/vice‐chairperson shall review and issue the approval letter. If the primary review opinion requires necessary revisions before approval, the chairperson/vice‐chairperson shall review and issue an ethics review opinion notification letter, which will be followed by the approval letter after the re‐review. The results of the expedited review shall be reported in the next regular meeting.
  If the primary review opinion is “approval,” the chairperson or vice‐chairperson reviews the findings and results, and issues the approval letter. In cases where the primary review opinion requires necessary revisions before approval, the chairperson or vice‐chairperson reviews the findings and results, and issues an ethics review opinion notification letter. Subsequently, an approval letter is issued after the re‐review process. The outcomes of the expedited review are to be reported in the next regular meeting.
7.3.4 The expedited review process may generally not exceed 14 working days.

## 8 CONFIRMATION METHODS

8.1 The institution is responsible for overseeing the operations of the ethics review committee and may delegate specific departments to conduct internal quality control of the committee's organization and functioning. The authorized department arranges for relevant personnel to conduct a self‐assessment of the committee's organization and management, compliance with laws and regulations, adherence to standard operating procedures, the ethics review process, follow‐up reviews, and document management. This self‐assessment should take place at least once a year.
8.2 The authorized department may perform evaluations using methods such as random sampling of relevant documents, interviews with key personnel (committee members, secretary, researchers' representatives, institutional administrators, etc.), and observation of meetings. The evaluation findings shall be documented in an evaluation report and provided as feedback to the ethics review committee.
8.3 Based on the identified issues, the ethics review committee shall develop improvement plans, which shall be reviewed and approved by the chairperson. The ethics review committee office is responsible for coordinating the implementation of the improvement work within the specified timeframe and documenting the completion status in writing. The progress and completion of the improvement work shall be reported to the ethics review committee meeting or the authorized department.
8.4 Documents shall be classified, managed, and preserved in accordance with archival requirements. Ongoing and completed projects should be filed separately, and each approved research project shall be labelled with a unique identifier. All documents should be arranged chronologically within folders, and labels and indexes should be created for easy reference. After a project is completed, the documents should be reviewed for completeness and archived in the project completion filing cabinet. The retention period should be maintained for 5 years after the research is completed.

## CONFLICT OF INTEREST STATEMENT

The authors declare no conflicts of interest.
